# Our Carolina Correspondent

**Published:** 1871-06

**Authors:** 


### PART III.

### Correspondence, Hygenic and Miscellaneous.

### OUR CAROLINA CORRESPONDENT.

DRS. POWELL & GOLDSMITH.—*Gentlemen:* I am well pleased —highly so—with the MEDICAL COMPANION AND ADVISER. The March number is excellent, well arranged and well illustrated with creamy articles and high-principled editorials. You are on the right track; you possess boldness and strike fearlessly. That's what we need—able advocates and fearless ones. I have been a subscriber for your State journals in the past, and am glad to say that no medical journal ever published in Georgia has ever surpassed it. In fact, it is far ahead of any before published on our soil in its scope and outspoken advocacy of truth, justice and right. I am glad to know, also, that your arrangements are of such a character as to give it permenency. It is bound to be a success; it meets the wants, precisely, of the practical and busy practitioner; it is a compendium; an "*omnium gathrum*" of all good things made ready for the hands of the go-ahead, active doctor; one who has not time to cull and gather; one who wants this service done for him. This you heve accomplished better and more admirably than any other journal in my knowledge. I do not desire to flatter; I speak the truth when I say the formulas you give in any one number are worth the subscription price. I would not do without this feature of your journal, and no practitioner can afford to do without it, if he will peruse it. I am gratified to see your enterprise so highly spoken of abroad. In my recent number of that excellent journal, the Baltimore Medical Journal and Bulletin, I notice the following complimentary language. It is true, and reflects deserved credit upon that journal, showing a fraternal liberality and kindness of spirit which all Southern men must applaud and commend. It says:

"The COMPANION is well gotten up, and promises well. We wish all success to the new enterprise. It is simply shameful if the South will not liberally sustain and encourage its own medical periodicals. In their efforts to advance the professional interests, there is no solid reason why, with a little fostering care, they may not take front rank in the medical literature of the country."

As a Southern man, this is my opinion. It would be "simply shameful"---a reproach upon the profession of your State, if they do not support your undertaking. If your journal should fail, the responsibility will fall upon them. I venture the assertion that it will not fail on your part—any one can see that. If it should, then the profession will prove unworthy of having a true exponent of its principles, and of having a diligent, busy worker ever at hand to open up for them the best medical thoughts of the times. You could only fail—not in matter and vim—but in neglecting to place it before the profession. They must know it to appreciate it, and if they know it they will. You may not be able to put it in the hands of all without pecuniary loss. The profession ought not to expect it. But, nevertheless, I beg leave to suggest, to get your publisher to send them for several months, to one, two or three hundred of the best medical gentlemen of the country—men who do appreciate good medical literature, and men who have zeal and energy in giving their influence to just such enterprises as yours. Let him appeal to them to lay it before the profession, and to secure their subscriptions to it. I will be one who will pledge myself for ten. I feel its necessity. I would not loose it for ten times the subscription price, and I believe the profession would not lose it if they could only feel its great value and importance.

